# Farmers' knowledge, practices and injuries associated with pesticide exposure in rural farming villages in Tanzania

**DOI:** 10.1186/1471-2458-14-389

**Published:** 2014-04-23

**Authors:** Elikana E Lekei, Aiwerasia V Ngowi, Leslie London

**Affiliations:** 1 Tropical Pesticides Research Institute, P.O. Box 3024, Arusha, Tanzania; 2 Department of Environmental and Occupational Health, Muhimbili University of Health and Allied Sciences (MUHAS), School of Public Health and Social Sciences, P.O. Box 65015, Dar es Salaam, Tanzania; 3 School of Public Health & Family Medicine - Faculty of Health Sciences, Anzio Road, Observatory 7925, Anzio, South Africa

**Keywords:** Farmers, Exposure, Knowledge, Pesticide poisoning, Associated injuries, Tanzania

## Abstract

**Background:**

Pesticides in Tanzania are extensively used for pest control in agriculture. Their usage and unsafe handling practices may potentially result in high farmer exposures and adverse health effects.

The aim of this study was to describe farmers’ pesticide exposure profile, knowledge about pesticide hazards, experience of previous poisoning, hazardous practices that may lead to Acute Pesticide Poisoning (APP) and the extent to which APP is reported.

**Methods:**

The study involved 121 head- of-household respondents from Arumeru district in Arusha region. Data collection involved administration of a standardised questionnaire to farmers and documentation of storage practices. Unsafe pesticide handling practices were assessed through observation of pesticide storage, conditions of personal protective equipment (PPE) and through self-reports of pesticide disposal and equipment calibration.

**Results:**

Past lifetime pesticide poisoning was reported by 93% of farmers. The agents reported as responsible for poisoning were Organophosphates (42%) and WHO Class II agents (77.6%).

Storage of pesticides in the home was reported by 79% of farmers. Respondents with higher education levels were significantly less likely to store pesticides in their home (PRR High/Low = 0.3; 95% CI = 0.1-0.7) and more likely to practice calibration of spray equipment (PRR High/Low = 1.2; 95% CI = 1.03-1.4). However, knowledge of routes of exposure was not associated with safety practices particularly for disposal, equipment wash area, storage and use of PPE . The majority of farmers experiencing APP in the past (79%) did not attend hospital and of the 23 farmers who did so in the preceding year, records could be traced for only 22% of these cases.

**Conclusions:**

The study found a high potential for pesticide exposure in the selected community in rural Tanzania, a high frequency of self-reported APP and poor recording in hospital records. Farmers’ knowledge levels appeared to be unrelated to their risk. Rather than simply focusing on knowledge-based strategies, comprehensive interventions are needed to reduce both exposure and health risks, including training, improvements in labeling, measures to reduce cost barriers to the adoption of safe behaviours, , promotion of control measures other than PPE and support for Integrated Pest Management (IPM).

## Background

Pesticide formulations distributed by licensed pesticide retailers are extensively used in Tanzania [1]. Over 13,000 metric tons (MT) of pesticide formulations were imported and distributed for use during 2003 and 2004 [2]. Given previous evidence of unsafe handling practices in Tanzania [3], the huge quantity of pesticides distributed suggests a high potential for human exposure, health injuries and illness. Indeed, a previous Tanzanian study identified acute pesticide poisoning (APP) as a major problem in the farming community [4].

At global level, it is estimated that hundreds of thousands of people die each year from the consequences of pesticides exposure [5,6] but the most problematic poisoning circumstance is suicide. Despite the high burden of APP in developing countries, there is substantial under-reporting suggesting that the burden of disease due to APP is frequently underestimated [7]. Studies in developing countries of farmer’s knowledge and practices have reported low to moderate levels of knowledge about pesticides [8,9], non-usage of personal protective equipment (PPE) [10,11], unsafe pesticide storage at homes [4,11], poor disposal of empty pesticide containers [8], misuse of pesticides and relatively low knowledge about pesticide safety labels [11]. A study on farmers’ safety practices in Ethiopia reported non usage or use of worn-out PPE [12].

These risks may be exacerbated by lack of information about the products handled. For example, some suppliers in Tanzania repackage and distribute products in unlabelled containers [13], and some distributors in Cambodia distribute products with labels written in a foreign language [14]. Studies in developing countries indicate that farmers usually source pesticide information from pesticide vendors and from other farmers [14] who are not knowledgeable about pesticide risks.

Previous research in Tanzania found that 68% of farmers reported episodes of feeling sick after routine application of pesticides and their pesticide-related health symptoms included skin problems and neurological symptoms [15]. However, the profile of pesticide products on the market has changed substantially in the decade since that study, which did not attempt to estimate the extent of under-reporting of APP. The aim of this study was therefore to describe farmers’ pesticide exposure profile, including their knowledge about pesticide risks, experience of poisoning and symptoms, and hazardous practices as well as the proportion of APP cases reported to health care facilities.

## Methods

The study site included agricultural areas cultivating coffee and vegetables in the Arumeru district in Arusha region. The site involved the villages of Uwiro, Olkung’wado, Nguruma, Moivaro, Makisoro, Ambureni and Sing’isi comprising about 5% of all villages in Arumeru district. The selected villages were typical of vegetable and coffee growers of Arumeru district. The target population was the heads of families and the sample size estimate of 130 was calculated using a margin of error of 8% with 95% Confidence and an a priori estimate from previous research in which 68% of farmers reported past APP [15].

Data collection involved administration of a questionnaire to farmers and observation of pesticide storage areas in the households visited. The questionnaire included both closed- and open-ended questions on past lifetime APP experienced, poisoning symptoms experienced at the time of poisoning, whether they attended a health facility, what action was taken after poisoning, practices regarding equipment calibration, storage and disposal, and knowledge about exposure routes. The farmers were asked to report the products associated with poisoning by trade names, which the majority of farmers were able to do. The corresponding active ingredients were accessed on the accompanied label or from the national list of registered pesticides. The association between exposure and symptoms was self reported by the respondents. Cases in which farmers claimed to have attended a health facility were tracked in health facility records to identify whether they were reported in the hospital systems.

The self reported data were collected by the PI, assisted by the trained local agricultural extension officers. Observation was conducted to verify farmer reports on pesticide storage and PPE availability for those farmers who reported having pesticides in storage on the day of the survey, which was about half (n = 57, or 47.1%) of the farmers. Their storage sites were also inspected for evidence of pesticide spillage. The data from farmers on self-reported storage location were compared with data on storage location recorded on physical inspection. For the rest of the households which were not inspected, data on storage location was based on what farmers reported.

The questionnaire was pre-tested among the farming community living near the Tropical Pesticides Research Institute (TPRI) offices in Arusha in January 2005 and found to capture the intended data. Data analysis was conducted using frequencies and percentages of all categorical variables. In bivariate analysis, variables were categorized as outlined in Table 1 below. Cut-offs used for dichotomising continuous variables were chosen as medians or close to medians in the distributions of respective variables. The product storage areas, use of PPE and disposal methods were categorised as safe or unsafe as specified in Table 1 below.

**Table 1:** Categorization of the data collected in household survey

**Data variable**	**Category**
**Storage area**	In house (defined as any of the following areas: bedroom, bathroom, toilet, kitchen, chicken-shed, above ceiling boards) or general store (store containing pesticides, fertilizers, food crops, farm implements and others)
	Other locations (defined as storage in pesticide stores or elsewhere on the farm)
**Education level**	High education (defined as ≥ form 4)
	Low education (defined as < form 4)
**Age**	Old (defined as >30 years)
	Young (defined as ≤30 years)
**Poisoning status**	Ever poisoned (defined as lifetime poisoning)
	Never poisoned (defined as a never experienced lifetime APP)
**Poisoning frequency**	Highly poisoned (defined as reporting poisoning frequency > 2)
	Not highly poisoned (defined as reporting poisoning frequency of ≤ 2)
**Product disposal**	Safe disposal (defined as safe burning, burying, dumping in a hole, re-spraying on field, donating to others or using up the pesticide)
	Unsafe disposal (defined as dumping in public disposal sites, on the farm, in the toilet or in the bush/ground)
**The use of PPE**	Users (defined as reporting current use of at least one form of PPE)
	Non-users (defined as reporting no current use of PPE)
**Pesticide container disposal**	Safe disposal (defined as safe burning or burying)
	Unsafe disposal (defined as re-use for household activities or dumping on the farm, in the toilet or in public sites)
**Gender**	Male (defined as male respondent)
	Female (defined as female respondent)
**Equipment calibration**	Yes (defined as respondents practicing calibration)
	No (defined as respondents not practicing calibration)
**Equipment washing area**	Close (defined as directly in the drinking water source or within 10 meters from the drinking water source)
	Other (defined as more than 10 meters away from the drinking water source)
**Knowledge on routes of exposure**	High knowledge (defined as reporting over 2 exposure routes)Low knowledge (defined as reporting ≤ 2 exposure routes)
**Steps taken after poisoning**	Health facility (defined as respondents attending health facilityl after poisoning) Other (defined as respondents not attending health facility after poisoning)

Cross-tabulations were conducted as follows:

(a) Poisoning frequency was compared by respondents’ education level, poisoning symptoms, the use of PPE, age, gender, practice of calibration, steps taken after poisoning, disposal practice and equipment wash area.

(b) Education level was compared by respondents’ practice including calibration, storage location and equipment wash area. Knowledge of routes of exposure was compared by disposal, use of PPE, calibration, equipment washing and education level.

(c) Lastly, poisoning status was compared by knowledge, education, use of PPE, calibration, equipment washing, storage and disposal.

Wilcoxon comparison of medians was used to test differences in medians for numeric data and Chi square (χ^2^) testing was used to compare the distribution of dichotomous variables. To measure the strength of association between categorical independent and dependent variables, Prevalence Risk Ratios (PRR) were estimated with 95% CIs. SPSS version 16 [16] and Stata Version 10.0 [17] were used to analyse the data.

To assess validity of responses regarding storage and PPE, reported responses were compared to observation data for those farmers whose premises were inspected (being farmers who reported having pesticides under storage at the time of survey; n = 57), and agreement between self-report and observation for indoor versus outdoor storage estimated as percentage of agreement. For validity of reporting of poisoning medical records for all respondents who reported pesticide poisoning in the past year and a random sample of 10% of those farmers who did not report APP in the past year, farmer medical records were traced in local health facilities in order to compare to their interview response. Validity was assessed as percentage of agreement.

The association between ever poisoning symptoms and poisoning frequency was tested for trend by calculating a Chi squared Mantel-Hantzel statistic.

A case of previous APP was defined as any self-reported short-term illness or health effects associated by the farmer with the preceding pesticide exposure. This approach has been used in other studies in developing countries [10,18,19].

Participants were fully sensitized on the study, and they completed a signed consent form before participation in the study and were free to decline participation without any consequence. To ensure confidentiality, names were replaced by codes for data analysis. The study protocol was approved by TPRI ethical committee and the National Institute of Medical Research (NIMR) in Tanzania (REF NIMR/HQ/Vol XI/371) as well as University of Cape Town (UCT) Health Science Faculty Research Ethics Committee (REF:328/2004).

## Results

A total of 121 farmers out of 130 participated in this study indicating a response rate of 93.0%. Most farmers were involved in cultivation of vegetables and coffee.

The respondents were mostly male (88.4%) and age ranged from 18–66 years with an average age of 37.5 years (SD 11.38). Most farmers had less than 7 years of education (88%).

Approximately 93% of respondents reported previous poisoning by pesticides in their lifetimes (past year inclusive) with frequency ranging from 1 to a maximum of 7 times; 76.4% of the poisoned respondents reported two or more poisonings and 63.5% reported 3 or more poisonings at some point in the past. The 112 farmers with past APP reported approximately 432 past poisonings in total. Actions taken after poisoning (not mutually exclusive) included drinking milk (25%), attending a health facility (21%), consulting a pharmacist (13%), applying cream to the affected area (6%), and washing the affected part of body (3%). However, most respondents (60%) reported taking no action following the poisoning. Of 23 farmers who reported attending health facility for poisoning in the past year, there were no records of their poisoning in health facility records for 18 cases (78.2%; 95% CI = 55.79%-91.71%). Overall, of farmers who claimed to have experienced a previous poisoning, 95.9% (95% CI 90.1-98.5%) did not appear in health facility surveillance records.

The active ingredients most commonly reported by farmers as associated with poisoning were Mancozeb (80%), Profenofos (72%), Chlorpyrifos (48%), Endosulfan (35%), Lambda Cyhalothrin (5%) and Cypermethrin (5%). Of the agents involved in reported poisonings, 42.4% were OP and 77.6% were moderately toxic products (WHO Class II). Among the products reported (n = 494) as handled by the farmers, 26% were OP pesticides, and 49% were WHO class II products (Table 2).

**Table 2:** Products reported as used by coffee and vegetable farmers in Arumeru district

**Active ingredient**	**Chemical group**	**WHO Class**	**Frequency (n = 121)**	**Percentage (%)**
**Copper**	Inorganic	III	68	56.2
**Endosulfan**	Organochlorine	II	66	54.5
**Mancozeb**	Dithiocarbamate	U	65	53.7
**Profenofos**	Organophosphate	II	60	49.6
**Chlorpyrifos**	Organophosphate	II	50	41.3
**Lambda Cyhalothrin**	Pyrethroids	II	25	20.7
**Abamectin**	Ivamectins	IV (EPA)*	23	19.0
**Others (Unclassified)**	-	-	21	17.4
**Cypermethrin**	Pyrethroids	II	16	13.2
**Unknown**	-	-	14	11.6
**Triadimefon**	Triazole	III	13	10.7
**Propineb**	Dithiocarbamate	U	13	10.7
**Metalaxyl**	Phenylamide	III	11	9.1
**Chlorothalonil**	Chloronitrile	U	10	8.3
**Dimethoate**	Organophosphate	II	10	8.3
**Deltamethrin**	Pyrethroids	II	9	7.4
**Fenitrothion**	Organophosphate	II	6	5.0
**Dip**	-	-	5	4.1
Others**			9	7.4

WHO Class Ia: Extremely hazardous, Ib: Highly hazardous.

II: Moderately hazardous, III: Slightly hazardous.

IV: Unlikely to present acute hazard under normal use condition.

*EPA – Environmental Protection Agency.

**Pirimiphos methyl (n = 3), Sulphur (n = 2), Novaluron (n = 2) and Amitraz (n = 2).

Lifetime Poisoning signs and symptoms reported as experienced by the farmers are listed in Table 3.

**Table 3:** Lifetime poisoning signs and symptoms (n = 875) self reported by coffee and vegetable farmers in Arumeru district

	**Sign/Symptom**	**Frequency (Farmers)**
1	Skin irritation	66
2	Chest pain	35
3	Coughing	34
4	Flu	65
5	Wheezing	14
6	Breathing with difficulty	40
7	Throat irritation	54
8	High fever	29
9	Excessive sweating	44
10	Nausea	34
11	Vomiting	6
12	Excessive salivation	43
13	Diarrhoea	10
14	Pain during urination	15
15	Stomachache	24
16	Tiredness	9
17	Nose bleeding	16
18	Blurred vision	42
19	Lacrimation	40
20	Eye irritation	61
21	Loss of appetite	21
22	Headache	66
23	Dizziness	49
24	Unconsciousness	10
25	Hands trembling	10
26	Sleepless nights	38
	**Total**	**875**

There were 875 symptoms associated with the 432 past poisonings reported by the 112 farmers.

In total, 81% of the respondents reported they kept pesticides within their residential homes, often in rooms used by a number of family members. Only 9% of respondents reported storing their pesticides in a dedicated pesticide store and 9% stored pesticides elsewhere on the farm (Table 4). The proportion of farmers self-reporting storage inside the home was 89.5% (95% CI = 77.8%-95.6%) in observed households and 68.8% (95% CI = 55.8%-79.4%) in households not observed. There was a high percentage of agreement (44/57, or 77%) between storage locations on self-report and observation on whether storage was indoors or outdoors.

**Table 4:** Reported storage locations, disposal methods, knowledge of routes of exposure and sources of pesticide information reported by coffee and vegetable farmers in Arumeru district

**Characteristic**	**Options**	**n**	**Percentage (%)**
**Storage of pesticides:**	General storage within the house	69	57.2
	Dedicated pesticide store	11	9.2
	Elsewhere on farm	11	9.2
	Toilet	7	5.9
	Kitchen	6	5.2
	Ceiling board	6	5.1
	Bedroom	4	3.0
	Bathroom	4	3.0
	Chicken shed	2	1.8
**Storage of spraying equipment:**	General store	47	39.0
	Equipment store	31	26.0
	Ceiling board	19	16.0
	Bedroom	16	13.0
	Elsewhere on the farm	8	6.4
Knowledge of pesticide absorption routes:*	Dermal	91	75.2
	Inhalation	88	72.7
	Ingestion	12	9.9
	Other (eyes, wound)	3	2.4
	Unknown	15	12.3
Farmers source of information on pesticides*	Label	86	70.8
	Extension officers	47	38.6
	Pesticides retailers	58	48.2
	TPRI	8	6.4
	Unknown	12	9.6
Disposal of unwanted pesticides*	Burn	4	3.3
	Bury	6	5.0
	Donate	5	4.1
	Dump in a hole	2	1.6
	Dump in general public sites including town disposal sites.	2	1.6
	Dump in the farm	35	28.9
	Dump on the ground	7	5.7
	Re spray remaining spray solution	42	34.7
	Dumping in the bush	2	1.6
	Dumping in the toilet	1	0.8
	Use all	11	9.0
	Do not Know	9	7.4

*Categories not mutually exclusive.

The main products found stored in the households at different locations were Mancozeb (n = 24), Chlorpyrifos (n = 19), Endosulfan (n = 13), Profenofos (n = 11) and Chlorothalonil (n = 10). Among the products found in the households (n = 99), 34.3% were WHO class II products. Table 5 shows the 10 most commonly cited active ingredients reported in three contexts: (a) as used, (b) stored and (c) associated with poisoning by the farmers.

**Table 5:** Ten most frequent active ingredients reported as used, stored and associated with poisoning among coffee and vegetable farmers in Arumeru district

**(a) Used**	**(b) Stored**	**(c) Associated with poisoning**
Endosulfan	Endosulfan	Endosulfan
Lambda cyhalothrin	Lambda cyhalothrin	Lambda cyhalothrin
Chlorpyrifos	Chlorpyrifos	Chlorpyrifos
Mancozeb	Mancozeb	Mancozeb
Cypermethrin	Cypermethrin	Cypermethrin
Profenofos	Profenofos	Profenofos
Abamectin	Abamectin	Abamectin
Copper fungicide	Copper fungicide	Malathion
Triadimefon	Amitraz	Triadimenol
Propineb	Chlorothalonil	Chlorothalonil

The *products* are not listed in order of descending frequency.

Containers reported as used for mixing pesticides included a special container for this purpose (80.2%), tractor mounted equipment (9.1%), backpack sprayers (5.8%) and containers also used for keeping drinking water (4.9%).

A wide variety of types of PPE was reported by farmers. The PPE most often used were gumboots (38.3%). Other reported PPE included long coats (n = 8), hats/helmets (n = 8), hand gloves (n = 6), overalls (n = 6), respirators (n = 6) and facemasks (n = 3). Most farmers (66.9%) reported no PPE use at all.

Of 40 farmers (33.1%) reporting PPE use, the number of different PPE items used ranged from 1 to 6. However, the quality and condition of the PPE were poor. Over 60% of the 117 PPE types reported among the 40 users were damaged or extremely contaminated when inspected. Most (4 out of 6) respirators reportedly used by the farmers were actually disposable dust masks unsuitable as PPE to prevent inhalation of pesticide droplets.

**Figure 1:**
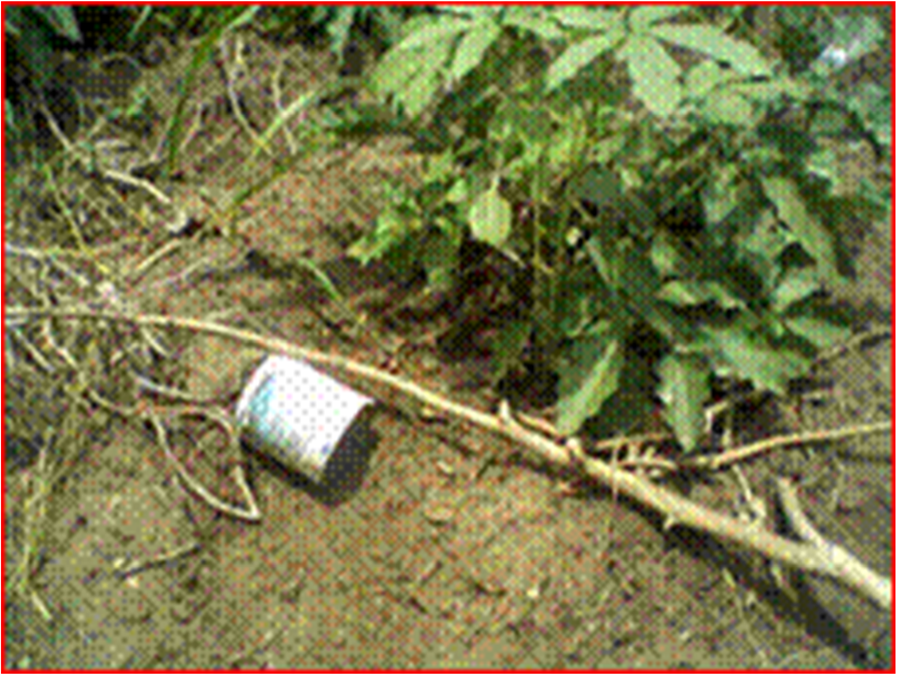
Dumping of pesticide containers in the farm demonstrating one of the unsafe disposal methods.

Methods of disposal of unwanted pesticides were reported as spraying on the crop (n = 42) and dumping out on the farm (n = 35) (Figure 1). Methods reported for disposal of empty pesticide containers included burying (n = 38), burning (n = 33), dumping on the farm (n = 25), selling back to pesticide retailers (n = 7) and reuse for household purposes (n = 8). Observation identified poor hygiene in the field with evidence that empty containers were not properly disposed of (Figure 1).

Farmers’ reported knowledge of the routes of absorption included mainly dermal (75.2%) and inhalational (72.7%) (Table 1). About 10% indicated lack of knowledge of any route of absorption.

Reported sources of pesticide handling instructions included pesticide labels (70.8%), pesticide retailers (48.2%) or agricultural extension officers (38.6%) (Table 3).

**Figure 2:**
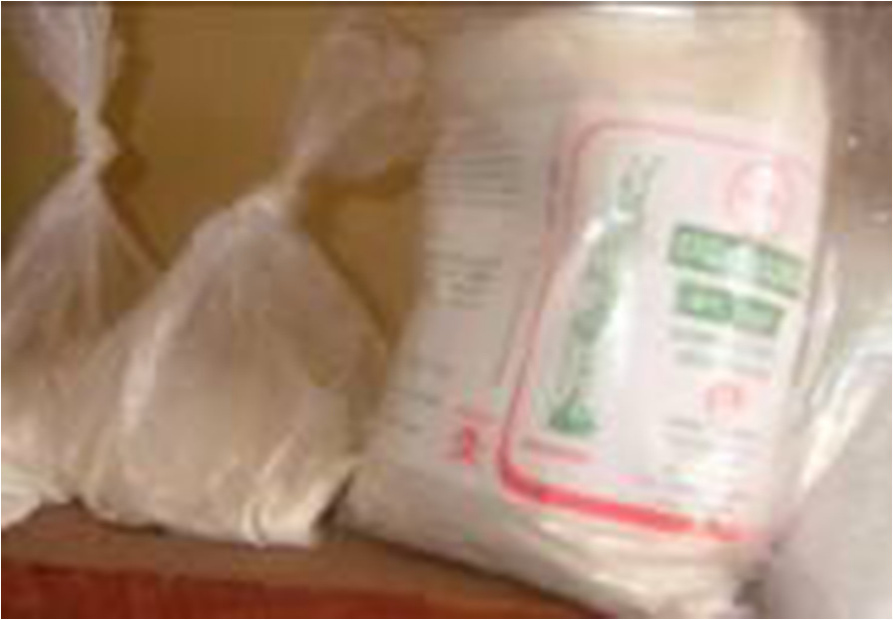
A pesticide (Endosulfan Dust) which is repackaged into a secondary container.

Among the 144 products found at 121 households, 36 products (25%) were found to have been repackaged into a secondary container. Of the 36 repackaged products, 42% were OP and 45% were WHO class II pesticides. The secondary containers were paper or plastic bags, glass or plastic bottles some of which were originally containers for drinking water or soft drinks (Figure 2).

Some of the products showed signs of spillage into surrounding surfaces. These agents included Copper Oxychloride, Copper Hydroxide, and WHO Class II agents namely Chloropyrifos, Pirimiphos Methyl and Profenofos. None of the repackaged containers had a proper label.

### Associations between frequency of past poisoning and high symptom reporting

There were marginally significant associations between high poisoning and: (i) washing spraying equipment close to water sources (PRR Close/Other = 1.2; 95% CI = 0.9, 1.6); and (ii) unsafe pesticide disposal practices (PRR Unsafe/Safe = 1.4; 95% CI = 0.9-2.4).

There was also a significant inverse association between high poisoning with storage of pesticides in living house (PRR Living house & Gen store/other: = 0.7; 95% CI = 0.6-0.9).

There were marginally significant associations between reporting high number of symptoms (over 10 poisoning symptoms) and a number of risk behaviours:(i) failure to use PPE (PRR Non-use/Use = 1.2; 95% CI = 0.9-1.6); (ii) failure to practice equipment calibration (PRR No/Yes = 1.2; 95% CI = 1.0-1.3); (iii) equipment wash area (PRR Close to water source/Other = 1.1; 95% CI = 0.9-1.5); (iv) equipment storage area (PRR Living house & general store/Other = 1.1; 95% CI = 0.9-1.3); (v) pesticide storage area (PRR Living house & general store/Other = 1.2; 95% CI = 0.9-1.4); and (vi) age (PRR Old/young = 1.1; 95% CI = 0.9-1.4).

The respondent’s knowledge showed no significant association with poisoning frequency and poisoning symptoms, respectively.

There was a significant association between storing pesticides in the house and respondents’ level of education. Respondents with high education were less likely to store pesticides in the home (PRR High/Low = 0.3; 95% CI = 0.1-0.7).

About 80% of the farmers reported that they did not calibrate their sprayers. There was a significant association between respondents’ knowledge (high knowledge versus low knowledge) and reporting practice of equipment calibration (PRR High/Low = 4.0; 95% CI = 1.3-12.8). Similarly, there was a significant association between respondents’ education (High education versus low education) and reported practice of equipment calibration (PRR High/Low = 1.2; 95% CI = 1.03-1.4).

## Discussion

Various sources of potential domestic and occupational pesticide exposure were noted during the survey. Firstly, the frequent storage of pesticides in homes indicated a high potential for exposure of farmers and family members due to storage in highly accessible places. Moreover, some of the products found stored within homes included moderately hazardous WHO Class II products such Chlorpyrifos, Profenofos and Endosulfan. Endosulfan is an organochlorine pesticide banned in many countries for health and environmental concerns and has been included in the list of POPs scheduled for elimination [20].

Storage of pesticides in unguarded sites in residences is common in many developing countries [3,21-23]. The prevalence of unguarded domestic storage was higher in this study (68%) compared to a previous Tanzanian study (43%) [3], probably because respondents in this study were not informed in advance of the researcher’s visit, so had no time to rearrange the stored products before the visit. Even so, the true prevalence of pesticide storage within households may have been slightly higher than reported given that the prevalence was higher (89.4%) in observed households compared to those where only farmer self-report was available (68.8%). However, agreement between observation and self-report in observed houses was generally high (77%) and the positive predictive value of self-reported indoor storage was 80%, suggesting that underreporting of household storage was not substantial and there was probably good validity for the self-report measure.

Secondly, failure to use PPE appeared to be another problem generating potential for significant pesticide exposure. This is supported by the finding of a significant association between high poisoning symptoms and non-usage of PPE (PRR Non usage/Usage = 1.3, 95% CI =1.0-1.6). Non-use of PPE might be caused by unavailability or high cost of PPE. The use of dust masks, which are relatively cheap, may suggest that farmers’ choices of PPE were influenced by considerations of minimizing costs. Similar findings about non usage of PPE amongst farmers have been reported in studies conducted in northern Greece [24] and India [25] and is often reported in other developing countries [2,18,22]. For example, Clark and colleagues [22] reported that, in the tropics, use of PPE is poorly tolerated because of discomfort associated with hot and humid conditions and prohibitive costs.

However, even when PPE was used, their protective role was limited in this study. For example, the most commonly reported PPE were gumboots, which were completely inadequate as sole protection. Also, the fact that farmers who reported use of respirators as protection were, on observation, mostly using disposable dust masks, which are not effective protection when spraying toxic pesticides, may paradoxically increase risk because the users mistakenly believe they are protected and so may not follow other safety precautions. Farmers who reported using respirators were not able to distinguish between a respirator and dust mask, highlighting the importance of measuring not only the presence but also the proper and appropriate use of PPE.

Two implications arise from these findings. Firstly, if studies do not examine the appropriateness of PPE used, the literature may over-report use of PPE. Secondly, had PPE use in this study been more appropriate, the association between failure to use PPE and symptoms may have been even stronger than that found in the study.

However, it is important to recognise that PPE too often it becomes a substitute for more important and sustainable safety measures, consistent with good occupational health and safety practice. For example, Integrated Pest Management (IPM), safer application methods and use of less toxic agents or mechanical barriers to pests are important ways to reduce reliance on, and, hence, human exposure to pesticides in agriculture. There is a hierarchy of controls in occupational health [26] in terms of which PPE should never be the first and only strategy. Further, it is important to acknowledge that PPE usage may be a proxy for safer practices in general. Farmers who use PPE may be more likely to practice better hygiene when handling pesticides, so the direct effect of PPE use may be difficult to establish from cross-sectional data. Longitudinal rather than cross-sectional studies would be better suited to testing this hypothesis.

Thirdly, equipment calibration is important to prevent both over-application, which results in human exposures, excessive residues and threats to local and export produce, as well as under-application, which may result in insect resistance. However, 80% of the farmers in this study did not calibrate their spraying equipment and appeared not to be conversant with the concept of calibration. The association between high poisoning symptoms reported by the farmers and failure to calibrate their equipment supports the argument that poor application practices can result in higher exposure through increased emission rates. Failure to calibrate equipment may similarly reflect poorer farmer hygiene practices in general and therefore be a proxy for other factors in farmers’ risk behaviour profile.

Fourthly, unsafe disposal of unwanted pesticides and empty pesticide containers may be an important source of pesticide exposure. Farmers commonly dumped products and containers in unsafe ways. These practices may lead to environmental contamination by runoff, leaching or distribution via aerial distribution to other areas and are typical of many developing countries [27]. About 5% of farmers indicated that they wash and re-use the empty pesticide containers for other household activities representing a route of serious non-occupational human exposure. A similar prevalence of re-use of containers has been reported in other studies in developing countries [27,28].

Prevalence of self-reported past poisoning among farmers was high (92.5%), higher than reported in Kenya [29], as was the frequency of poisoning episodes (61.1% of farmers reporting 4 or more previous poisonings). These figures most likely reflect non-severe cases which go unrecorded in the absence of an APP surveillance system because they do not present to health facility. Such APP cases might be best captured in community-based self-reporting systems.

A number of findings showing the importance for surveillance emerge in this study. The most obvious is that 60% of poisoned respondents did nothing about their symptoms, and 81% did not report going to a health care facility. Poisoned farmers may not report their injuries to a health care facility for a number of reasons including (i) inability to afford payment for their medical bills; (ii) the majority of poisonings being of mild severity; (iii) anticipated difficulties in diagnosis and treatment deter attendance; (iv) distance to health care facility or poor access to health services and, (v) anticipation of lack of appropriate drugs or medical services in the majority of the health facilities. Poisoned farmers may also be unaware of the long term adverse health effects of pesticides, further contributing to a lack of motivation to attend health facilities. This means that facility-based surveillance is likely to miss poisoning cases among farmers who do not access services for their poisoning. Even when attending, cases may not be recorded in hospital databases due to poor recording systems. In this study, 18 of the 23 farmers who reported attending a health facility due to poisonings in the past year could not be traced in medical records at the facilities they claimed to have attended. This suggests a large proportion of cases presenting to health facilities (78.2%) are unreported in hospital information systems and an even larger proportion (95.9%) of all farmers who claimed to have experienced a previous poisoning (both those who attend and do not attend facilities for the poisoning) are unrecorded in hospital-based surveillance due to under-reporting or misclassification particularly because the symptoms were not specific. The extent of under-reporting is almost exactly the same as that found in a South African study (95%) of APP cases unreported in the Western Cape Province in 1994 – 1995 [30].

Under-reporting has also been documented in Nicaragua (approximately two thirds of APP) [31,32]. The finding of low reporting of APP in this study is consistent with other community-based studies with farmers in both developing [29] and developed [7] countries, reporting that a minority of poisoned farmers (between 8% and 25%) seek health care. This has important implications for surveillance, which is key to prioritizing and evaluating interventions to control the problem. Strategies to ensure that the full spectrum of cases of pesticide poisoning is captured by surveillance are therefore urgently needed, particularly in developing countries where the exposures and risks are highest.

Of the 10 pesticides most commonly reported as causes of poisoning (Table 5), most (70%) were also listed as most commonly used or stored in households. This suggests a consistent link between pesticide distribution and subsequent human exposure, and also points to the value of data on distribution of pesticide ingredients as a potentially useful form of surveillance as a proxy for exposure.

Also of note, is that at least 5 agents commonly responsible for poisoning in this study (lambda-cyhalothrin, chloropyrifos, cypermethrin, endosulfan and profenofos) were previously reported in Tanzania [3] as causes of pesticide poisoning. In contrast, in this study, DDT which is banned for agricultural use was not reported, whereas Mancozeb was not previously reported but was present in this study, reflecting a change in use pattern of products over time in Tanzania. Similarly, WHO class I products were not reported as major causes of poisoning in this study, most likely because they are now registered for “restricted use” in Tanzania and are therefore unlikely to be used by small scale farmers. Restriction of highly toxic pesticides has been shown to be a successful strategy in reducing mortality in Sri Lanka [33].

Among the specific active ingredients associated with poisoning in this study, OP’s (42.4%) and class II agents (77.6%) accounted for the highest proportions. The contribution of OPs may be underestimated because some unknown agents may have been OPs.

One reason for the lack of complete consistency between products reported as used and those seen on inspection is that the farmers might have been out of stock during the survey and hence some products could not be found at home. Secondly, some of the farmers may not be conversant with the products they handle and, as a result, they may fail to report correctly all pesticides in use. In this study, 12.3% of farmers failed to report all the products they handle. On the other hand, underreporting may also be the result of poor recordkeeping by some farmers.

Endosulfan, which was widely reported in this study, and found stored within homes, belongs to the group of persistent organic pollutants (POPs). It has already been banned in 56 countries because of its high toxicity and environmental impact [34]. In terms of acute toxicity, endosulfan is highly toxic to aquatic life [35] and there have been a number of human deaths associated with endosulfan exposure, particularly in Africa and India [36]. An intervention study in Sri Lanka showed that after an endosulfan ban in 1998, over a 3 -year period following the ban, deaths due to APP fell from more than 15-fold in the selected district hospitals [33]. In terms of chronic toxicity, endosulfan is an endocrine-disruptor, mimicking oestrogen at very low levels of exposure and is implicated in breast cancer. It is also a neurotoxin and has been linked to Parkinson's disease, birth defects and immunotoxicity [35]. Endosulfan has been associated with developmental and reproductive effects in children environmentally exposed on cashew nut plantations in India [33]. Based on this accumulating evidence base, in October 2008, the Review Committee of the PIC met and concluded that endosulfan met the criteria for inclusion in the PIC (Rotterdam) treaty. Despite this, several countries exporting the pesticide, including India, blocked its addition to the prior informed consent (PIC) schedule [37]. This study, therefore, provides additional evidence to support the inclusion of endosulfan on the PIC list.

The frequency of use of OP and WHO Class II pesticides (28% and 49%, respectively) in this study is lower than previously reported (64% and 76% respectively) by a Tanzanian farmers’ study [3]. The previous study was conducted during 1991 – 1993, and the differences observed may be due to changing trends in Tanzanian agricultural practices with the introduction of newer products, particularly pyrethroids.

Most of these products are used on export crops [38]. This trend is mirrored by similar shifts in the pattern of agents most commonly reported as causing poisoning. Also, some farmers may use alternative pest control methods such as Integrated Pest Management (IPM) that reduce reliance on more toxic chemical pesticides. IPM measures introduced in Tanzania after 1993 include the use of airtight drums for storage [39], botanicals and inert materials such as dust, cow dung and ashes to protect harvested maize and neem seed powder, pyrethrum dusts and synergized pyrethrum for storage pests in general [37] and the use of pheromones to trap insects in the field [40].

In general, the protective effect of higher levels of formal education and knowledge of pesticides among the farmers was modest. High educated farmers and farmers with high knowledge were more likely to report practicing equipment calibration (OR = 1.2; 95% CI 1.03-1.4, and OR = 4.0; 95% CI 1.3-12.8, respectively) and high-educated farmers were less likely (OR= 0.3; 95% CI 0.1-0.7) to report storing pesticides in their homes. Farmers with low education and low knowledge would be expected to have less awareness of the health and environmental implications associated with pesticides and more inclined to store pesticides in their homesteads.

It is possible that farmers may have acquired their knowledge after being poisoned, in that increased symptoms led to both increased awareness and less willingness to store pesticides in the home. This may explain the counterintuitive finding that storing pesticides in the home was inversely associated with high poisoning since the data collected on storage was for current practice while poisoning was for past events. Neither PPE usage nor knowledge was associated with the frequency of past poisoning. There may also be some underreporting of poisoning due to reporting bias with reported hygiene practices not reflecting the real situation, and there may be other routes of exposure which were not measured in this study. Despite this, the findings suggest that there may be benefits for the prevention of poisoning with better education and awareness.

Nonetheless, it is still clear that for many safety practices, both education and, particularly, knowledge appeared to play no role. In particular, there was no association in the use of PPE with either education or knowledge. Yet, the study also demonstrated that farmers were not ignorant of the potential health effects or routes of absorption of pesticide, with over three-quarters of farmers reporting awareness of the main routes of absorption. Similar findings of good knowledge have been reported by Clark, et al. [22] in Ghana. This suggests that even though farmers may know very well the hazards of the chemicals they work with, there may be other social and economic factors beyond their control that increase their risk of poisoning. For example, farmers may be well aware of the hazards but adopt risky practices like unsafe storage and omission of PPE use because of economic pressures to increase production or disincentives related to the costs of PPE and safe storage.

Interventions that provide farmers with information should therefore be coupled with other economic and social strategies to make hygiene practices economically and practically feasible. Of concern, though, is the misconception reported by 25% of farmers that milk could serve as an antidote following poisoning. This was the single most commonly reported action taken after a pesticide poisoning. This myth appears to be widespread among farmers and workers in diverse settings in developing countries [41,42] and particularly persistent, despite the lack of evidence for its efficacy. In a study conducted in Tanzania, 64.7% of agriculture extension officers reported recommending milk as a first aid measure in pesticides poisoning [43] so the fact that farmers are poorly informed may reflect information they are receiving from multiple supposedly trustworthy sources.

Farmers in this study appeared to rely heavily on the labels (69%) as their main source of information and, to lesser extent, on extension officers and pesticide retailers. This reliance on labels as a major source of information is similar to findings in a study in Vietnam where 65% of farmers reported relying on pesticide labels as a source of information [44]. However, this source of information is of limited quality since many labels are damaged to the extent that they could not be easily read or understood by the users. The situation was more serious for the products distributed in non-original containers like soft drink containers which bear no relevant information. Further, some pesticides may have the correct labels but the information may not be understandable to the users due to use of complicated terminology or language.

Farmers’ reliance on labels for information on pesticides may reflect the fact that the proliferation of pesticide suppliers under trade liberalization policies in Tanzania [9], which facilitated an 80-fold increase in the number of unregulated suppliers in the 1990s, resulting in the involvement of children in pesticide retailing as well as insufficient technical support for small farmers.

It is particularly worrying, given evidence of poor comprehensibility of labels for working populations in developing countries [45-48] and the reliance on labeling contained in the new system for Global Harmonization of Chemical Hazard Classification and Labeling (GHS) being introduced by the United Nations [45-48]. Extension officers are expected to fill this gap but there are currently too few to meet demands for the farming community. Also, some of the available extension officers, as reported in a previous Tanzanian study [43], are not adequately trained on pesticide health aspects. Training of the extension officers is therefore strongly recommended.

Pesticide retailers who supply the products to the farmers are also a potential source of information for farmers on pesticide handling, given the inadequate number of agriculture extension officers in many areas. However, many retailers were not well trained in safe handling of pesticides and are likely to be biased in promoting the sale of their products at the expense of empowering farmers to make independent decisions about pest control methods. They may encourage farmers to use their products over non-chemicals methods or products from other companies, which may create confusion among the farmers.

This study found that few farmers disposed of their empty pesticide containers through returning them to manufacturers. Returning of empty containers to manufacturers could be a useful method for safe and economic disposal, but, in Tanzania, there is no direct link between a farmer and the manufacturer. Communication happens almost exclusively through product distributors or retailers who have no incentive to recycle containers. Another limitation is that some retailers may misuse the empty containers for decanting or repacking of adulterated products instead of returning them to manufacturers. The manufacturers have no policy for the collection of empty pesticide containers from the farmers. Moreover, if farmers were to sell the empty containers back to pesticide retailers as a means of disposal this could be detrimental for safety, particularly with unscrupulous retailers, because it could create a market for empty containers and hence encourage product adulteration through repacking and decanting. This, in turn, may lead to the distribution of substandard products to the farmers.

Handling of repackaged, decanted as well as spilled products, observed in this study, are highly hazardous practices, and probably the result of distribution of pesticides in large containers, which are unaffordable for small scale farmers. Instead, small-scale farmers who have modest needs for pesticides on their small size farms will purchase small amounts decanted into secondary containers including soft drink bottles, which are particularly hazardous.

The use of containers for refilling pesticides is another potentially unsafe approach for disposal, due to the fact that it can encourage product adulteration and movement of products with misleading instructions or with no instructions at all leading to poor handling and application. Although only a few cases of reuse of empty containers for domestic purposes (4.9%) were noted in this sample of farmers, the situation is prevalent in many developing countries. Studies in Madhya Pradesh, India [49], Tanzania [3] and South Africa [28] found that rural populations made use of empty pesticide containers for domestic purposes, such as for keeping domestic water.

### Study limitations

The main limitation in this study was the use of self-report to define a case of APP. Although this is commonly applied in many countries, the approach is likely to overestimate the burden caused by pesticides exposure.

Another limitation in this study was the limited generalisability arising from non-random sampling and potential bias introduced during the selection of the sample. It is possible the 7 villages from which participants were drawn are systematically different from villages in other parts of Tanzania; certainly, the crop production differs to other parts of the country and the villages’ previous relationship to the TPRI may make them more sensitive to pesticide safety issues. However, if farmers had underreported their hazardous practices, the findings would have underestimated the extent of the problem. A different study conducted in Tanzania [15] found similar age and educational levels among farmers in a different part of northern Tanzania, suggesting that the sample was unlikely to differ very much from similar types of farmers in Tanzania.

Another problem may be an expectation of incentives (financial or other) for research participants, based on farmers’ previous experience of large foreign-funded research projects. The absence of any compensation may have discouraged some of the farmers from participating. Conversely, farmers with past histories of pesticide poisoning may have been more likely to participate. Nonetheless, the extent of non-participation was low (less than 10%) so was unlikely to make a big difference to the findings.

Thirdly, there were also potential information biases. Social interaction among respondents who belong to a common social group was experienced in a few situations such that they responded by providing similar answers. Once detected, participants were interviewed separately to avoid cross-communication. Further, poor knowledge about pesticides among the respondents, such as the failure to identify a pesticide product by its trade name or common name and classification, might have contributed to misreporting of poisoning agents or increased the number of poisonings due to unknown agents (42.8%). The problems due to OP and due to WHO Class I and II pesticides may therefore be substantially underreported.

Farmers’ responses about poisoning symptoms, especially past poisoning events and past products handled, may have been subject to poor recall if details were forgotten. Despite having some awareness of hazards and routes of exposure, farmers may have been unable to link all symptoms to particular exposure. This might have led to underestimation of the reported association of OP-related poisoning symptoms and OP products handled.

Additionally, the low farmer awareness of signs and symptoms consistent with pesticide poisoning is likely to create confusion in differentiating APP symptoms and symptoms from other diseases conditions. This appeared to be a general problem and probably contributed to over-reporting of APP symptoms and the inclusion of symptoms arising from causes other than pesticides. However, underreporting may have resulted from exclusion of APP symptoms due to failure to recognize them. The nett effect may have cancelled out any misclassification but further studies would be needed to clarify the relative effects. Moreover, despite the limitations listed above, the test for trends in poisoning frequency against poisoning symptoms was statistically significant (Data available from the authors; Chi square Mantel Hantzel p < 0.05).

Another limitation is that the poisoning impact of the agents reported was evaluated using WHO classification based on active ingredient. Because different formulations of the same active ingredient may have different toxicity, there may have been imprecision in the estimates by WHO class.

Also as pointed out, the associations identified were based on cross-sectional data which provides weak evidence for causality. Longitudinal studies would be needed to provide stronger evidence for the causality of associations identified in this study.

Lastly the reporting of APP as associated with the products of low toxicity such as Malathion might be an indication either of over-reporting of APP by the farmers or symptoms caused by the co-formulants rather than the Active ingredient itself.

## Conclusion and recommendations

This study has revealed potential opportunities for human and environmental exposure to pesticides in a selected community in rural Tanzania. Although based on a non-random sample, the farmers in this study appear typical of farmers in rural Tanzania. This suggests a potentially serious public health problem that may be widespread in the country. The study findings are also important in contributing to advocacy for sound interventions especially with decision-makers in Tanzania who are currently considering amendments to the Plant Protection Act of 1997 [50]. The findings can also be used to contribute to the establishment of a national surveillance system for APP.

Interventions are needed to improve pesticide storage conditions at local level and to ensure surveillance strategies that capture all the poisoning cases, including those that do not present to health care facilities. Efforts to develop community monitoring [23] should be supported.

Farmers in this study had reasonably good knowledge about routes of exposure and hazards but had poor safety practices, particularly for disposal, equipment calibration, storage and use of PPE. To some extent, these are safety practices that require practical knowledge for implementation, although costs may be prohibitive. Training of the farmers on safety practices is recommended but should be practically-oriented involving farmer field schools because evidence shows that these schools are the most effective ways to change farmer behaviour [51]. Moreover, training should be complemented by measures that reduce cost barriers to the adoption of safe behaviours.

Where provided, training must address adverse health effects associated with pesticide exposure, safe handling and reading and interpretation of pesticide label instructions, which were found to be a major source of information to the farmers in this study. Label instructions should be written in simple language, easily understood by the user taking into account the requirements of the National Law in Tanzania [50] and the Globally Harmonized System for Chemical Hazard Classification and Labeling (GHS) [47].

Training and oversight of pesticide retailers by the National Authority through programs that are currently in place is also critical to ensuring safety along the supply chain. Training of farmers on the use of control measures other than PPE should be stressed since some farmers seem to rely on PPE as the only control measure for exposure. In principle, PPE should be the last resort in the hierarchy of control measures. Many of the above measures will not be effective without adequate enforcement. Finally, the issue of farmers’ unsafe practices, found in this and many other studies, is complex because it involves interventions to change farmer behaviour. Although recommendations made here seek to address this problem, further qualitative studies need to be done to address this issue in a comprehensive manner.

## Competing interests

The authors declare that they have no competing interests.

## Author’s contributions

EEL: Carried out the study design, field survey, data collection, analysis and results interpretation. He also drafted the manuscript and was the corresponding author. AVN: Participated in the statistical data analysis and supported drafting and revision of the manuscript. LL: Participated in the study design, statistical data analysis and supported interpretation. He was also involved in the drafting and revision of the manuscript. All authors read and approved the final manuscript.

## Pre-publication history

The pre-publication history for this paper can be accessed here:

http://www.biomedcentral.com/1471-2458/14/389/prepub
